# Acoustic divergence in advertisement calls among three sympatric *Microhyla* species from East China

**DOI:** 10.7717/peerj.8708

**Published:** 2020-03-11

**Authors:** Zhi-Qiang Chen, You-Fu Lin, Yun Tang, Guo-Hua Ding, Yan-Qing Wu, Zhi-Hua Lin

**Affiliations:** 1 Laboratory of Amphibian Diversity Investigation, College of Ecology, Lishui University, Lishui, Zhejiang, P.R. China; 2 College of Animal Science and Technology, Zhejiang A & F University, Lin’an, Zhejiang, P.R. China; 3 College of Forestry, Nanjing Forestry University, Nanjing, Jiangsu, P.R. China; 4 College of Life Sciences, Nanjing Normal University, Nanjing, Jiangsu, P.R. China; 5 Nanjing Institute of Environmental Sciences, Ministry of Ecology and Environment, Nanjing, Jiangsu, P.R. China

**Keywords:** Acoustic differentiation, Body size, Intraspecific variation, Microhyla, Species recognition, Phylogenetic effect

## Abstract

**Background:**

Species-specific advertisement calls are the main mechanism of transmitting information between individuals in anuran amphibians and are therefore indispensable for anuran survival and reproduction. Survey methods that monitor these calls can be used for rapid species recognition, behavioral experiments, and conservation monitoring. In this study, we described in detail 10 call parameters from three sympatric species in the genus *Microhyla* and analyzed the differences in call parameter among these species to provide a basis for systematic monitoring, acoustic analysis and taxonomic study of this genus.

**Methods:**

The quantitative analyses of temporal and spectral call parameters were used in our study for the advertisement calls of three sympatric *Microhyla* species (*M. beilunensis*, *M. fissipes* and *M. heymonsi*) in Zhejiang Province, East China.

**Results:**

Our results showed the following: (1) Significant differences existed among the three sympatric *Microhyla* species in call duration (CD), call interval (CI), number of pulses (NP), pulse rate, call intensity (CIT), dominant frequency (DF) and frequency of the first to fourth formants (F1, F2, F3 and F4). (2) Some spectral parameters (DF, F1 and F3) were negatively correlated with the body size of the vocalizing individuals in each species. (3) The coefficients of variation within individuals (CV_w_) for CIT, DF and F1–F4 were smaller than 5%, whereas the CV_W_ for CI was larger than 10% in each species. (4) The principal component analysis and discriminant function analysis showed that call parameters could distinguish the three *Microhyla* species. (5) The phylogenetic generalized least squares analysis showed that phylogenetic relationships affected CD and NP against snout-vent length (SVL), DF and NP against CD, and NP against DF, but not of DF against SVL; based on the phylogenetic analysis, CD and NP were not related to SVL, but DF was negatively related to SVL.

## Introduction

Vocalizations, whose functions are mainly involved in reproduction, aggressive behavior and defense, is one of the important mode of communication in anuran amphibians (Gerhardt & Huber, 2002; Kelley, 2004; Wells, 2007; Toledo et al., 2015b). Advertisement calls are a form of male anuran vocalization that is most commonly heard and of the highest value in taxonomy and they play a crucial role in attracting potential mates and conveying territorial information to conspecifics. Several researchers have focused on advertisement calls of anurans (Wells, 2007; Gambale, Signorelli & Bastos, 2014; Köhler et al., 2017; Poyarkov et al., 2019; Wei et al., 2019). The variation in advertisement calls in anurans is generally studied at the intraspecific and interspecific levels (Köhler et al., 2017). Call variation within and between individuals of many anurans is influenced by individual motivation of the vocalizing male owing to numerous internal and/or external factors (Wells, 2007; Köhler et al., 2017). For example, social context could change the frequency of calls (Bee, Perrill & Owen, 2000; Reichert & Gerhardt, 2013) and temperature effects are linked to pulse rate and call duration (CD) (Lingnau & Bastos, 2007; Pröhl et al., 2007; Gasser, Amézquita & Hödl, 2009; Bee, Suyesh & Biju, 2013; Ziegler, Arim & Bozinovic, 2015) at the individual level. Furthermore, body size could constrain the frequency of calls (Gerhardt & Huber, 2002; Gingras et al., 2013) and physical or physiological damages could generate differences in calls (Hoffmann & Kloas, 2012; Pröhl et al., 2013) among individuals. Interspecific variation in calls is generally considered for species recognition in anurans (Gerhardt & Huber, 2002) and it is higher than intraspecific variation (Köhler et al., 2017). Therefore, Köhler et al. (2017) suggested that the assessment of variation in advertisement calls in anurans is important for understanding speciation and signal evolution.

Several studies on advertisement calls in anurans have focused on the variation pattern in spectral and temporal parameters (Gerhardt, 1991; Bee, Reichert & Tumulty, 2016). The static and dynamic properties of call parameters are proposed to be relevant to species recognition and mate choice, respectively (Gerhardt, 1991). In most anuran species, spectral and fine temporal call parameters are relatively stable (more static) at the within-individual level, whereas gross temporal parameters represented higher variability (more dynamic) (Gerhardt, 1991; Reinhold, 2009; Köhler et al., 2017). When a parameter has a relatively low variation, it is hypothesized to be caused by stabilizing selection of female preferences (Gerhardt, 1991; Castellano & Rosso, 2006). However, the static and dynamic traits of call parameters are not strictly inalterable, that is, these traits of a specific call parameter may vary among anuran species (e.g., dominant frequency (DF), Gambale, Signorelli & Bastos, 2014; Wei et al., 2019).

**Figure 1:**
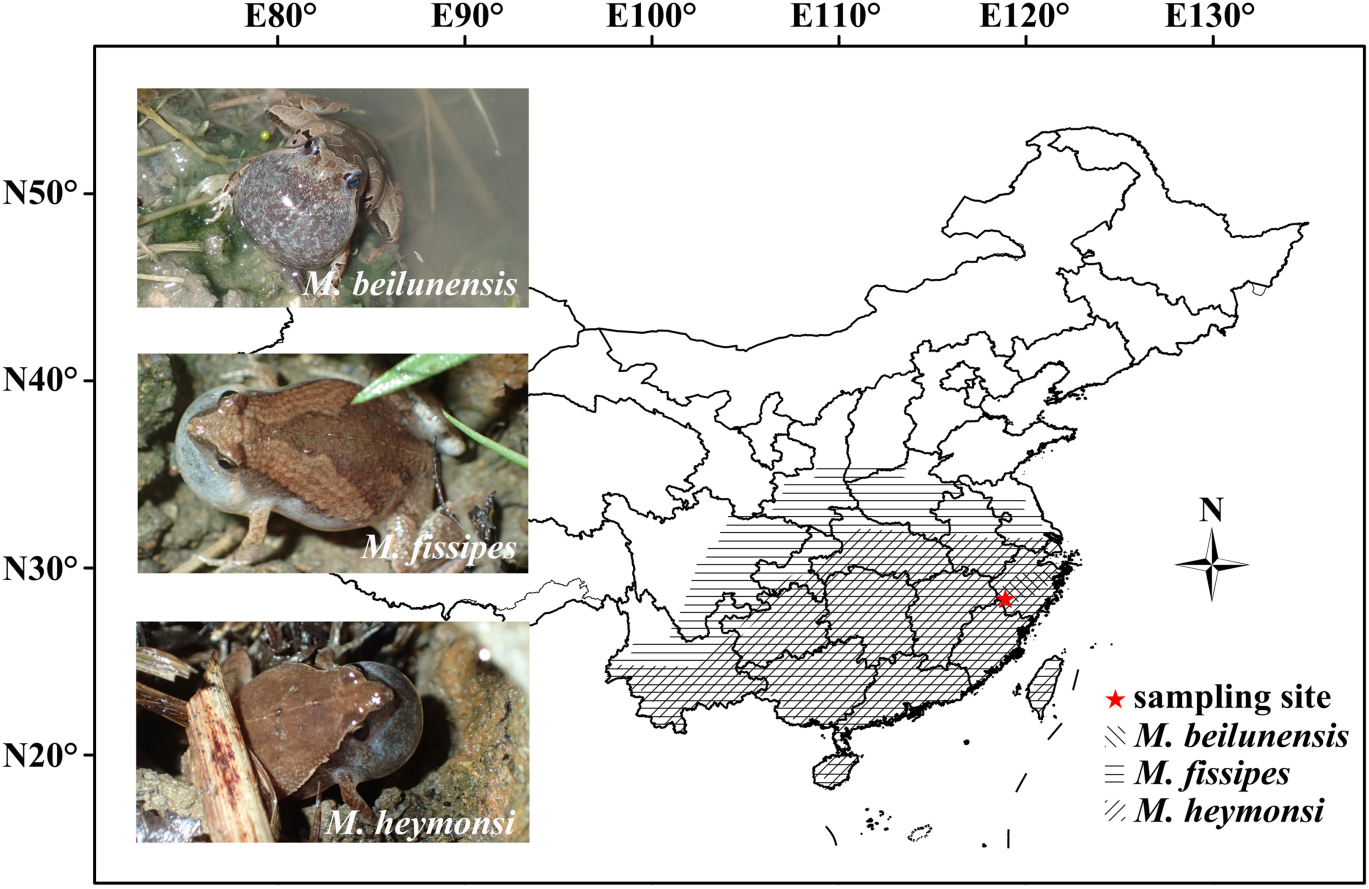
The geographical distribution of the three *Microhyla* species in China. Photos of the three *Microhyla* calling males in field (Photo by Guo-Hua Ding) and sampling site were showed in the figure.

*Microhyla* (Anura: Microhylidae) is a genus with 50 species that are distributed from the Ryukyu Island and South China through India to Sri Lanka and through Southeast Asia to Sumatra, Borneo, Java and Bali (Frost, 2020). Nine species of *Microhyla* are currently known from Southeast China (AmphibiaChina, 2020). To date, there are reports on the advertisement calls of 28 *Microhyla* species (Table S1). Several studies have reported the taxonomy and identification of *Microhyla* species in some distribution areas (Sri Lanka, Myanmar and South Asia) through bio-acoustic comparison (Wijayathilaka, Meegaskumbura & Gianni, 2016; Garg et al., 2018; Poyarkov et al., 2019). In East China, *M. beilunensis* Zhang et al. (2018), *M. fissipes* Boulenger (1884) and *M. heymonsi* Vogt (1911) are three common *Microhyla* species and their distribution ranges are overlapping (Fig. 1). They breed from April to June in shallow, ephemeral pools and paddy flied (AmphibiaChina, 2020; Fei, Ye & Jiang, 2012; Z.-Q. Chen et al., 2019, unpublished data). In this study, we quantified call parameters in these three species. The results will be of importance for systematics, rapid identification of species, behavioral experiments and species protection. Particularly, they will be useful to identify species quickly and accurately in field surveys. We quantified the spectral and temporal parameters of advertisement calls in the three sympatric *Microhyla* species in the Jiulongshan National Nature Reserve, Lishui City, Zhejiang Province, East China. We also explored the relationships among snout-vent length (SVL), CD, number of pulses (NP) and DF using previously published data, while accounting for phylogenetic relationships.

## Materials and Methods

### Data collection

Administration Bureau of Jiulongshan National Nature Reserve provided the permit for capturing animals in the field (No. 20180422). Our experimental procedures complied with the current laws on animal welfare and research in China and were specifically approved by the Animal Research Ethics Committee of College of Ecology in Lishui University (Permit No. ARE-CECLU 201804-001).

The advertisement calls of males from three *Microhyla* species were recorded in the Jiulongshan National Nature Reserve (28.37°N, 118.90°E), in April and May 2017–2018. Males of *M. fissipes* and *M. heymonsi* were captured in the same six paddy fields, while males of *M. beilunensis* were captured from four paddy fields adjacent to those of other two species. Each frog was individually placed in a lidded, transparent, plastic cage (20 cm × 10 cm × 10 cm) and the cage was then placed on the ridge of the paddy field, where the frogs were initially collected. After placing the cage for 10 min, the call of each male was recorded for at least 1 min at 20:00–22:00 h, using a Sony IC recorder (ICD-SX1000) with an internal microphone, placed at 0.5 m from the cage, with a sampling frequency resolution of 44,100 Hz and 16-bit resolution. Thereafter, the SVL was measured using a digital Mitutoyo caliper (±0.1 mm). A total of 1,328 valid advertisement calls were recorded successfully, from the 45 males (17 *M. beilunensis*, 13 *M. fissipes* and 15 *M. heymonsi*), across all three species (444 calls of *M. beilunensis*, 475 calls of *M. fissipes* and 409 calls of *M. heymonsi*). We also recorded the ambient air temperature during the experiment, using the UNI-T digital thermometer (UT325). The air temperature during the experiment ranged between 16.8 °C and 18.2 °C (for *M. beilunensis*) and between 16.3 °C and 19.8 °C (for *M. fissipes* and *M. heymonsi*). After recording, all individuals were released.

### Bioacoustics analysis

Recordings were transferred from the recording device to PC with Cool Edit Pro 2.1. Then, background noise was reduced by 20 dB (FFT = 1,024 points) in the noise reduction module and files were saved in the “.wav” format. Advertisement calls were analyzed using Praat 6.0.49 (Boersma & Weenink, 2019) at a sampling frequency resolution of 44,100 Hz and 16-bit resolution. The temporal and spectral structures of the calls were analyzed using 10 variables: CD, call interval (CI), NP, pulses rate of the call (PR), call intensity (CIT), DF and the frequency of the first to fourth formants (F1, F2, F3 and F4).

### Data analyses

We used coefficients of variation within individual (CV_W_) and among individuals (CV_A_) to quantify the variability in call parameters. CV_W_ was calculated as the standard deviation (SD) divided by the mean, for each acoustic parameter and for each individual. An overall mean CV_W_ was calculated for each species and for each parameter. CV_A_ was calculated for each parameter using the overall mean and SD, for all individuals of the same species (Pettit, Bourne & Bee, 2013). With respect to CV_W_, the parameters with a mean that explained up to 5% of the total variance were considered static. The parameters with a mean of between 5% and 10% were intermediate and those with a mean that explained more than 10% of the total variance were considered dynamic (Gerhardt, 1991). The magnitude of sample variation was calculated using the ratio of CV_A_/CV_W_ for each parameter; a non-parametric Kruskal–Wallis *H* test was used to evaluate if CV_A_ was higher than CV_W_ (Morais et al., 2012; Gambale, Signorelli & Bastos, 2014; Turin, Nali & Prado, 2018).

Calls were pooled by individual specimens. Statistical analyses of the calls were conducted using SPSS 18.0 software. Prior to parametric analysis, the normality and homogeneity of variance in the data were tested using the Kolmogorov–Smirnov test and Bartlett’s *M* test, respectively. Data did not require any transformation to meet the assumptions of the parametric tests. We used Pearson’s correlation analyses (correlation coefficient = ρ) to assess the relationship between SVL and the 10 call parameters, for each species. We used min–max normalization to homogenize the call variable data for each species and carried out principal component analysis (PCA) to determine the relative contribution of each of the 10 acoustic parameters to call distinctiveness. We used discriminant function analysis (DFA) to verify the results of the PCA. A call parameter was classified as the main contributing factor if its absolute value was higher than 0.7 (Turin, Nali & Prado, 2018). One-way ANOVAs was used to determine the difference in acoustic characteristics between the three *Microhyla* species, with factor scores for the two axes. Tukey’s post hoc test (HSD) was carried out on variables that differed among the three species. All values are presented as mean ± SD and the differences are considered statistically significant at α = 0.05.

We combined our data with previously published records of the SVL, CD, NP and DF of advertisement calls from 29 *Microhyla* species (Table S1). The tests detailed previously were carried out using the topology including the 29 *Microhyla* species collected. The species topology was based on phylogenetic relationships (Fig. S1). For phylogenetic analyses, we downloaded the mitochondrial 12S rRNA, 16S rRNA and CO I sequence data for the related species from GenBank (Table S2). Concatenated sequence data of 12S (351 bp), 16S (508 bp) and CO I (577 bp) of the 29 *Microhyla* species were used for phylogenetic reconstructions. According to Matsui et al. (2011), one *Kaloula verrucosa* sample was chosen as the outgroup. Phylogenetic relationships were reconstructed using the Bayesian Inference (BI) method, implemented in MrBayes v. 3.2 (Ronquist et al., 2012). Before that, the best-fit substitution model was selected using jModeltest v. 2.1.4 (Darriba et al., 2012) under the corrected Akaike’s Information Criterion (AIC; Hurvich & Tsai, 1989). Based on the results, the GTR+I+G model was selected for the BI phylogenetic analyses. In BI, we initiated a dependent run with four simultaneous Monte Carlo Markov chains (MCMC) for 20 million generations with sampling every 1,000 generations and discarded the first 25% of generations as burn-in after confirming the convergence of chains. The final majority tree and posterior probabilities (pp) were achieved from the remaining trees. We used substitutions/site of BI method to represent branch length. Ordinary least squares (OLS) and phylogenetic general least squares (PGLS) regressions were implemented in R 3.2.3 (R Development Core Team, 2015), using the RMS (Harrell, 2012) and CAPER (Orme et al., 2012) packages. OLS regression was used to estimate the slope for all conventional analyses. PGLS regression was used to examine the relationships between pairs of variables (SVL, CD, NP and DF), while accounting for phylogenetic effects. PGLS regression incorporates phylogenetic information into generalized linear models and offers a powerful methodology for analyzing continuous data. Previously, it has been applied to estimate the evolutionary relationship between traits of interest (Warne & Charnov, 2008; Barros, Herrel & Kohlsdorf, 2011; Yu et al., 2014). In PGLS regression, the strength and type of phylogenetic signal in the data matrix can be accounted for by adjusting branch length, which can be optimized to find the maximum likelihood transformation. We used AIC to estimate merits and drawbacks of the models tested; the best model was that with the lowest AIC and λ, using a maximum likelihood approach to evaluate phylogenetic effects (λ = 0 indicates no phylogenetic effect and λ = l indicates the strongest phylogenetic effect equivalent to that expected under the Brownian motion model). The best-fitting model can be determined using the maximum-likelihood ratio test.

## Results

**Figure 2:**
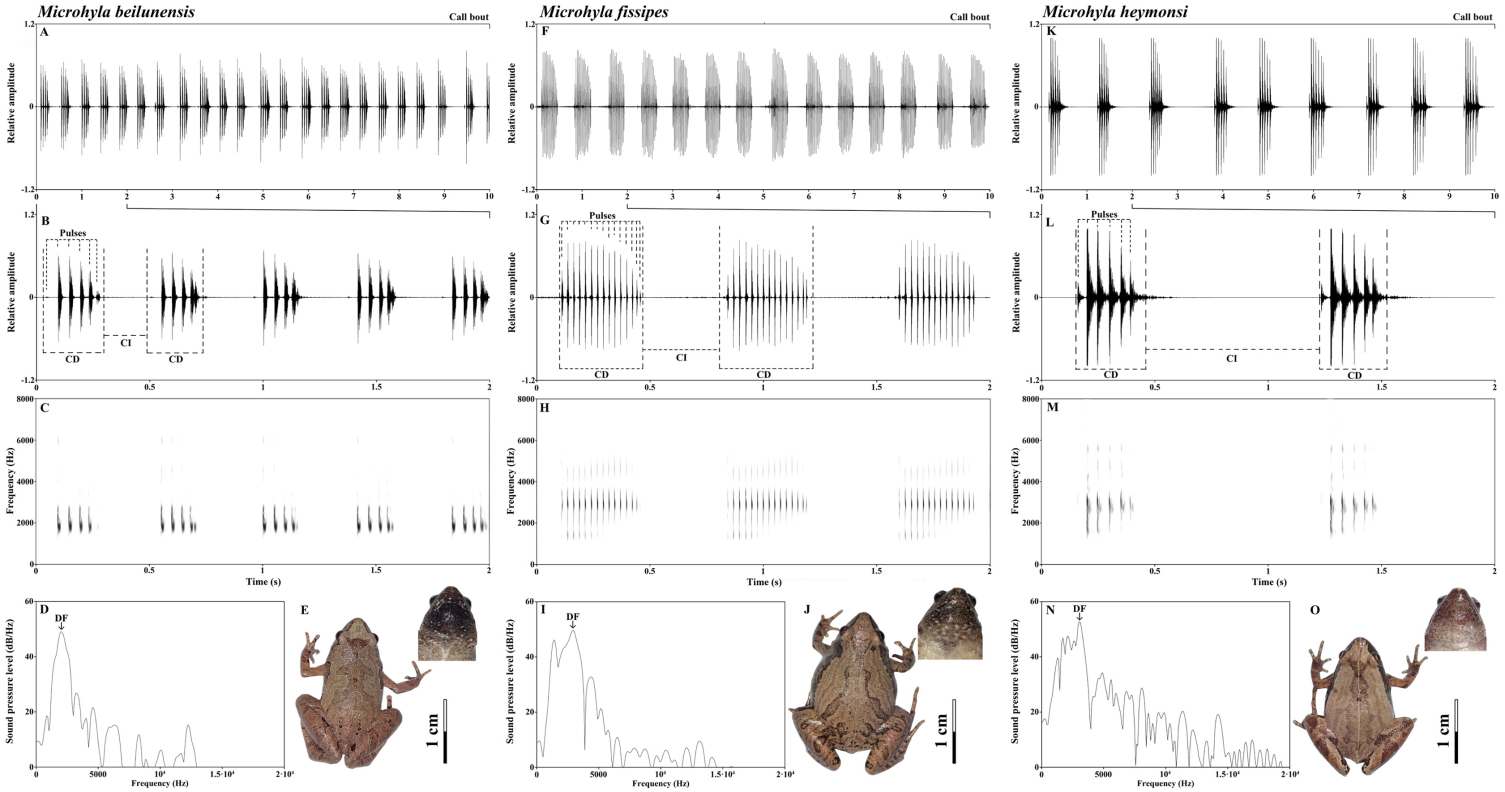
Sample recordings of the advertisement call of the three *Microhyla* species from East China. (A, F and K) Oscillogram of a 10 s long call bout of the three *Microhyla* species recorded in Suichang, Zhejiang, China (B, G and L) detailed view of a 2 s section of the oscillogram and (C, H and M) spectrogram of the same call (D, I and N) power spectrum of the same advertisement call, and (E, J and O) dorsal and pharyngeal views of males in M. *beilunensis*, *M. fissipes* and *M. heymonsi*, respectively. *M*. *beilunensis* is characterized by a brown triangular marking between the eyes, dark-brown markings with light brown margins on the dorsum and limbs and a brown-black pharyngeal; *M. fissipes* is characterized by a pink-gray, yellow-brown or gray-brown dorsum, two inverted V-shaped marking tandem lined on the dorsum and a black pharyngeal; *M. heymonsi* is characterized by a yellow thin ridge line from snout to vent, a pair or two pairs of brown arcuate spots on the ridge line of dorsum and brown stripes on the pharyngeal (AmphibiaChina, 2020; Zhang et al., 2018).

The advertisement calls of all three species had approximately the same spectrogram shape: first a pulse of low amplitude, which then increases, then the main body of the call, then a final pulse, during which the amplitude decreases again. It is a spindle-shaped trend in which the amplitude gradually increased, and then gradually decreased (Fig. 2). However, there were species-specific differences in call composition (Fig. 2). All species exhibited significant differences in the frequency of the NP. The call of *M. beilunensis* was composed of 2–7 separate pulses (Figs. 2B and 2C); a six-pulse call occurred most frequently, comprising 37.70% of the total calls. The call of *M. fissipes* had 8–21 separate pulses (Figs. 2F and 2G); the 16-pulse call occurred most frequently, comprising 33.75% of thetotal calls. The call of *M. heymonsi* was composed of 3–11 separate pulses (Figs. 2K and 2L); the six-pulse call occurred most frequently, comprising 43.40% of the total calls. Males of all three species have an external single subgular vocal sac and their throat color differs among the three species (Figs. 2E, 2J and 2O). *M. beilunensis* has small black markings around the throat (Fig. 2E). The throat of *M. fissipes* has large black markings on all sides and small black markings in the center (Fig. 2J) and that of *M. heymonsi* has small brown markings (Fig. 2O).

The mean SVL differed among the three species (*F*_2, 42_ = 11.51, *P* < 0.001) and was the largest in *M. fissipes* (23.2 ± 1.2 mm; ranged, 21.6–25.8 mm; *N* = 13) and the smallest in *M. heymonsi* (21.5 ± 1.2 mm; ranged,19.6–23.6 mm; *N* = 15), with *M. beilunensis* (22.5 ± 0.7 mm; ranged, 21.2–23.5 mm; *N* = 17) in between. The correlation analysis between SVL and the 10 call parameters showed that SVL negatively correlated with the DF in *M. fissipes* (ρ = −0.698) and *M. beilunensis* (ρ = −0.652), with the F1 in *M. heymonsi* (ρ = −0.558) and *M. beilunensis* (ρ = −0.494) and with the F3 in all three species (*M. beilunensis*: ρ = −0.553, *M. fissipes*: ρ = −0.733, *M. heymonsi*: ρ = −0.526).

All the 10 call parameters differed significantly among the three species (Table 1). The CD, DF, F2 and F3 were higher in *M. fissipes* and *M. heymonsi* than in *M. beilunensis*; the CI and CIT were the highest in *M. heymonsi* and the lowest in *M. beilunensis*; the NP was the highest in *M. fissipes* and the lowest in *M. beilunensis*; the PR was higher in *M. fissipes* than in *M. heymonsi* and *M. beilunensis*; and the F1 and F4 were lower in *M. fissipes* than in the other species (Table 1).

**Table 1:** Descriptive statistics, expressed as means ± SD and range, for acoustic characteristics of advertisement calls in the three *Microhyla* species from East China. Results of one-way ANOVAs are given in the table. CD, call duration; CI, call interval; NP, number of pulses; PR, pulse rate; CIT, call intensity; DF, dominant frequency; F1, the first formant; F2, the second formant; F3, the third formant; F4, the fourth formant; MB, *M. beilunensis;* MF, *M. fissipes*; MH, *M. heymonsi*.

Call parameters	Species			Statistical results
	*M. beilunensis*	*M. fissipes*	*M. heymonsi*	
*N*	17	13	15	
CD (s)	0.273 ± 0.027	0.334 ± 0.030	0.308 ± 0.046	*F*_2, 42_ = 11.33, *P* < 0.001
	0.248–0.331	0.286–0.375	0.248–0.386	MF = MH > MB
CI (s)	0.238 ± 0.072	0.483 ± 0.095	0.919 ± 0.098	*F*_2, 42_ = 241.99, *P* < 0.0001
	0.126–0.387	0.325–0.595	0.762–1.068	MH > MF > MB
NP (pulses)	5.0 ± 0.4	14.8 ± 1.7	6.3 ± 0.8	*F*_2, 42_ = 354.56, *P* < 0.0001
	4.4–5.6	11.3–16.6	5.3–7.6	MF > MH > MB
PR (pulses/s)	18.7 ± 2.2	44.9 ± 7.3	20.1 ± 1.5	*F*_2, 42_ = 163.85, *P* < 0.0001
	15.1–22.3	30.6–55.7	19.4–24.7	MF > MH = MB
CIT (dB)	74.6 ± 0.9	75.5 ± 1.3	79.0 ± 0.4	*F*_2, 42_ = 96.15, *P* < 0.0001
	72.8–76.1	73.0–77.5	78.5–79.8	MH > MF > MB
DF (Hz)	2,139.2 ± 218.9	2,951.5 ± 151.8	2,877.5 ± 188.4	*F*_2, 42_ = 107.60, *P* < 0.0001
	1,750.1–2,411.7	2,644.8–3,116.8	2,696.7–3,048.3	MF = MH > MB
F1 (Hz)	1,642.9 ± 52.1	1,472.3 ± 52.4	1,589.0 ± 44.0	*F*_2, 42_ = 44.28, *P* < 0.0001
	1557.3–1,750.7	1,353.7–1,530.3	1,630.3–1,662.4	MF < MH = MB
F2 (Hz)	2,122.1 ± 132.5	2,336.1 ± 62.1	2,389.7 ± 70.7	*F*_2, 42_ = 33.98, *P* < 0.0001
	1,848.9–2,326.7	2,256.1–2,437.2	2,275.3–2,512.4	MF = MH > MB
F3 (Hz)	2,651.0 ± 71.7	2,963.4 ± 117.6	2,951.2 ± 90.9	*F*_2, 42_ = 57.16, *P* < 0.0001
	2,544.2–2,827.5	2,715.7–3,080.0	2,842.4–3,129.9	MF = MH > MB
F4 (Hz)	3,805.9 ± 63.7	3,641.9 ± 135.2	3,803.9 ± 68.0	*F*_2, 42_ = 14.80, *P* < 0.0001
	3,723.7–3,936.5	3,315.0–3,934.8	3,714.8–3,917.3	MF < MH = MB

In all the species, four temporal parameters (CD, CI, NP and PR) showed larger CV_W_ and CV_A_, whereas six spectral parameters presented smaller coefficients (Table 2). The mean CV_A_ was larger than the mean CV_W_ for all the parameters (the ratio of CV_A_/CV_W_ > 1.0), and these differences were statistically significant (Table 2).

**Table 2:** Descriptive statistics of coefficients of variation within (CV_W_) and among individuals (CV_A_) CV_A_/CV_W_ ratio and statistical comparison between CV_W_ and CV_A_ (Kruskal–Wallis *H*) in the three *Microhyla* species from East China.

Call parameters	*M. beilunensis*					*M. fissipes*					*M. heymonsi*				
	CV_W_ (%) (range)	Property	CV_A_ (%)	CV_A_/CV_W_	*H*	CV_W_ (%) (range)	Property	CV_A_ (%)	CV_A_/CV_W_	*H*	CV_W_ (%) (range)	Property	CV_A_ (%)	CV_A_/CV_W_	*H*
CD	14.7 (8.1–23.1)	Dynamic	18.19	1.24	13.55*	7.3 (4.6–12.4)	Intermediate	11.30	1.54	9.07*	24.4 (11.1–41.9)	Dynamic	31.44	1.29	7.45*
CI	41.7 (15.1–59.7)	Dynamic	52.04	1.25	13.96*	27.5 (10.8–53.1)	Dynamic	35.47	1.29	9.07*	26.6 (17.8–41.5)	Dynamic	28.53	1.07	6.92*
NP	18.9 (9.1–29.9)	Dynamic	20.52	1.09	13.21*	8.6 (5.1–15.1)	Intermediate	13.58	1.59	9.07*	20.1 (8.7–43.4)	Dynamic	25.58	1.28	7.88*
PR	15.5 (10.6–23.2)	Dynamic	19.28	1.25	13.96*	5.5 (2.7–8.3)	Intermediate	16.72	3.06	9.92*	12.2 (5.9–26.4)	Dynamic	14.72	1.20	7.45*
CIT	1.3 (0.6–2.1)	Static	1.86	1.39	13.96*	0.9 (0.3–2.4)	Static	1.98	2.22	9.07*	1.1 (0.5–1.7)	Static	1.20	1.12	6.92*
DF	4.1 (0.4–8.0)	Static	11.25	2.71	15.71*	2.3 (0.9–6.1)	Static	5.26	2.26	9.07*	3.0 (0.3–9.0)	Static	5.47	1.82	8.41*
F1	3.7 (0.8–5.8)	Static	5.04	1.36	13.55*	1.1 (0.4–3.4)	Static	3.40	3.01	9.92*	2.2 (1.1–3.4)	Static	3.52	1.60	9.80*
F2	2.9 (0.9–5.8)	Static	6.92	2.36	15.71*	1.2 (0.5–3.7)	Static	2.93	2.47	9.07*	2.9 (0.6-5.0)	Static	4.20	1.43	7.88*
F3	2.9 (0.9–6.1)	Static	4.25	1.44	13.55*	1.3 (0.2–5.0)	Static	4.17	3.14	9.07*	1.9 (0.6–3.2)	Static	3.44	1.83	9.80*
F4	3.2 (0.8–8.4)	Static	4.10	1.29	13.55*	2.1 (0.4–5.5)	Static	4.38	2.10	9.07*	2.8 (1.8–5.5)	Static	3.27	1.18	7.45*

**Notes:**

* *P* < 0.01.

CD, call duration; CI, call interval; NP, number of pulses; PR, pulse rate; CIT, call intensity; DF, dominant frequency; F1, the first formant; F2, the second formant; F3, the third formant; F4, the fourth formant; CV_W_, Within-individual coefficients of variation; CV_A_, Among-individual coefficients of variation; Static, CV_W_ ≤ 5%; dynamic, CV_W_ ≥ 10%; intermediate, 5% < CV_W_ < 10%.

**Figure 3:**
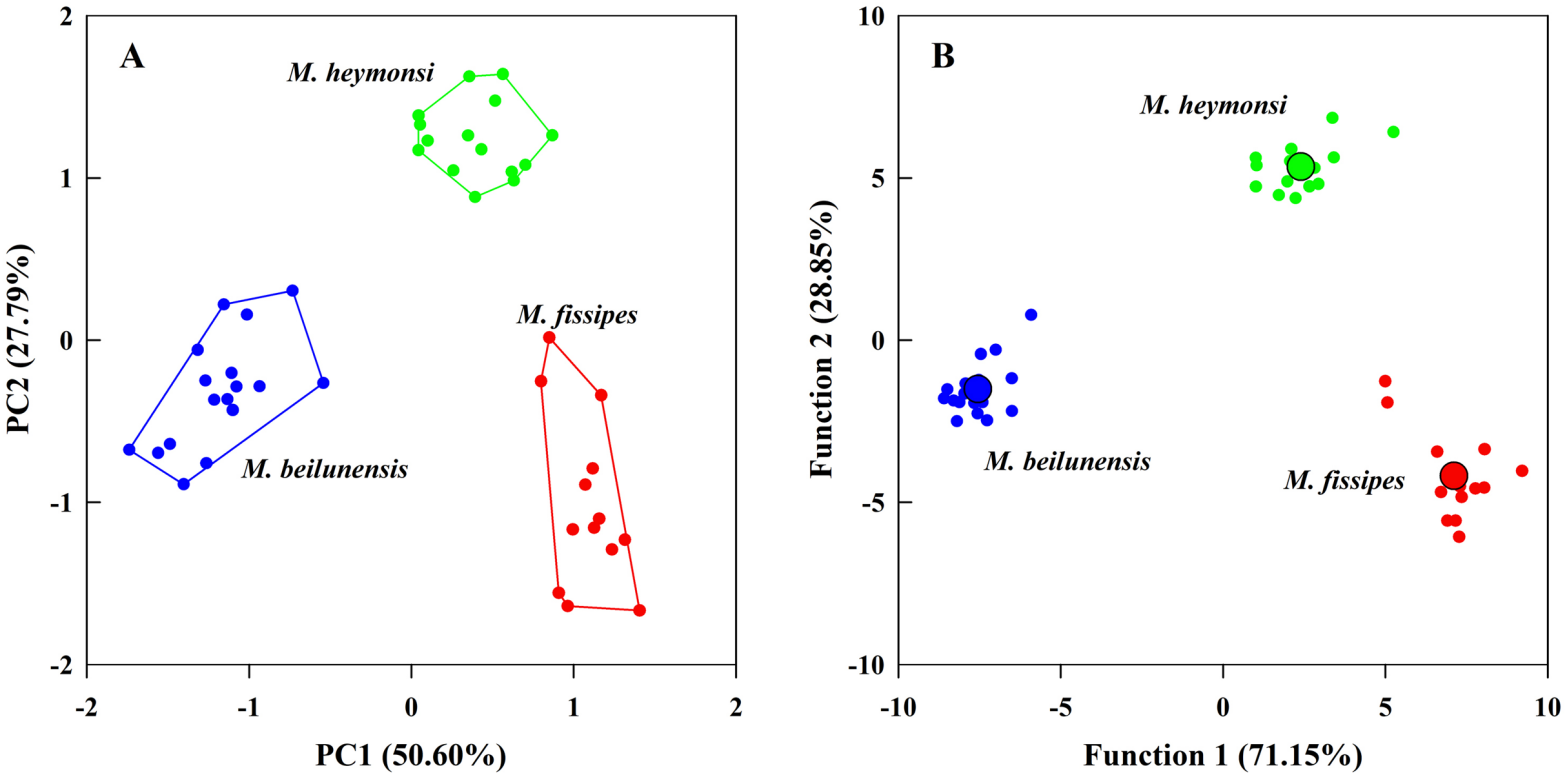
The principal component analysis and the discriminant function analysis of advertisement call parameters for the three *Microhyla* species. (A) Plot of principle components 1 and 2 (PC1 vs. PC2) based on ten variables of acoustic parameters. (B) Canonical variables plot of the discriminant function analysis based on 10 variables of acoustic parameters with the centroids indicated as enlarged dots.

The PCA analysis suggested that two components (eigenvalue ≥ 1) from the original ten acoustic parameters accounted for 78.39% of the variation (Table 3). The first component (explained 50.60% of variance) had high positive loading scores for NP, PR, F2 and F3 and a high negative loading score for F1; the second axis (explained 27.79% of variance) mainly represented CI and CIT (Table 3). The resulting scores differed significantly among the three species on both axes (first axis: *F*_2, 42_ = 323.24, *P* < 0.0001; MH > MF > MB; second axis: *F*_2, 42_ = 131.73, *P* < 0.0001; MH > MB > MF) (Fig. 3A).

**Table 3:** Factor loadings of the Principal Component Analysis (PCA) and standardized coefficients of the Discriminant Function Analysis (DFA) on the advertisement call parameters of the three *Microhyla* species from East China. Absolute values higher than 0.7 are shown in bold. CD, call duration; CI, call interval; NP, number of pulses; PR, pulse rate; CIT, call intensity; DF, dominant frequency; F1, the first formant; F2, the second formant; F3, the third formant; F4, the fourth formant.

Call parameters	PCA		DFA	
	PC1	PC2	Function 1	Function 2
CD	0.637	−0.112	0.013	0.687
CI	0.548	**0.751**	0.620	**0.713**
NP	**0.791**	−0.585	**0.964**	**−1.365**
PR	**0.734**	−0.598	−0.183	**1.035**
CIT	0.433	**0.789**	0.182	0.309
DF	**0.939**	0.255	0.200	−0.486
F1	**−0.701**	0.414	−0.571	0.159
F2	**0.794**	0.434	−0.238	0.489
F3	**0.856**	0.283	**0.741**	0.372
F4	−0.517	0.613	−0.045	−0.333
Eigenvalue	5.059	2.782	40.798	16.546
Variance (%)	50.59	27.82	71.15	28.85
Cumulative variance (%)	50.59	78.41	71.15	100

The DFA analysis presented results similar to those of the PCA and generated two discriminant functions (Table 3). The first canonical variable (explained 71.15% of variance) mostly represented NP and F3; the second canonical variable (explained 28.85% of variance) was largely represented by the CI, NP and PR (Table 3). The two functions differed significantly among the three species (Wilks’ λ = 0.001, *P* < 0.0001); even after excluding the first function, the second function was still different for each species (Wilks’ λ = 0.057, *P* < 0.0001). The centroids were −7.55, −1.52 for *M. beilunensis*; 7.11, −4.18 for *M. fissipes*; and 2.40, 5.34 for *M. heymonsi* (Fig. 3B).

**Figure 4:**
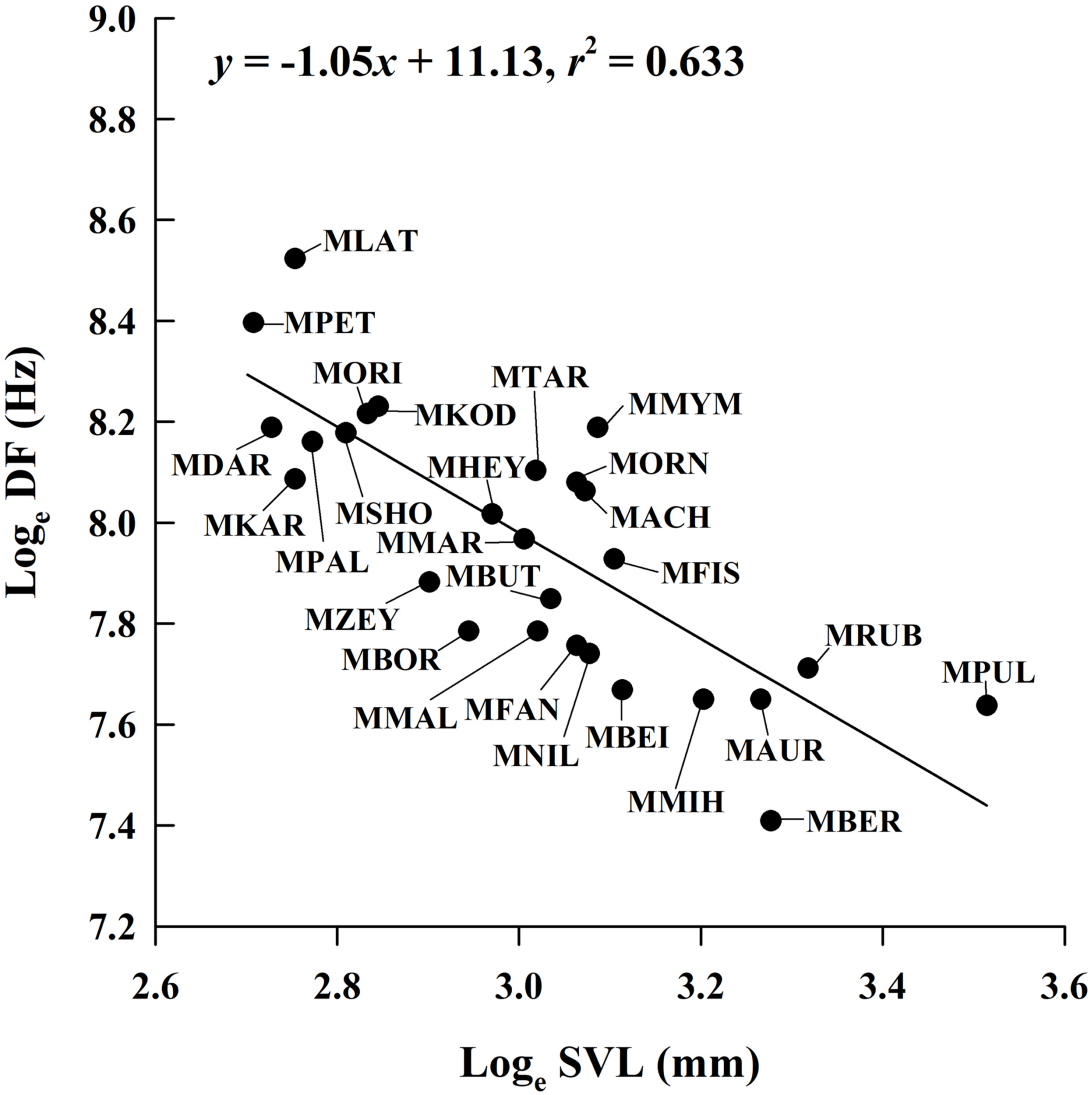
Ordinary least squares regression of dominant frequency (DF) on male snout-vent length (SVL) of 26 *Microhyla* species. Regression equation and coefficient are given in the figure. MACH, *M. achatina*; MAUR, *M. aurantiventris*; MBEI, *M. beilunensis*; MBER, *M. berdmorei*; MBOR, *M. borneensis*; MBUT, *M. butleri*; MDAR, *M. darreli*; MFIS, *M. fissipes*; MFAN, *M. fanjingshanensis*; MHEY, *M. heymonsi*; MKAR, *M. karunaratnei*; MKOD, *M. kodial*; MLAT, *M. laterite*; MMAL, *M. malang*; MMIH, *M. mihintalei*; MMYM, *M. mymensinghensis*; MNIL, *M. nilphamariensis*; MORI, *M. orientalis*; MORN, *M. ornata*; MPAL, *M. palmipes*; MPET, *M. petrigena*; MPUL, *M. pulchra*; MRUB, *M. rubra*; MSHO, *M. sholigari*; MTAR, *M. taraiensis*; MZEY, *M. zeylanica*.

By incorporating data from 29 *Microhyla* species, we found that the mean male SVL in the genus ranged from 14.9 mm to 33.6 mm; mean CD ranged from 0.062 s to 1.806 s; mean NP ranged from 4 to 97; and mean DF ranged from 1,650 Hz to 5,029 Hz (Table S1). The PGLS analysis showed that phylogenetic relationships affect CD vs. SVL, NP vs. SVL, DF vs. CD, NP vs. CD and NP vs. DF (all λ > 0.77), but not DF vs. SVL (λ = 0) (Table 4). DF was negatively related to SVL (Fig. 4), but the relationship between CD and SVL, NP and SVL, DF and CD, and NP and DF were not significant in the OLS and PGLS models (Table 4). NP was positively related to CD in the OLS model, but not in the PGLS model (Table 4). Based on likelihood ratio tests, the PGLS model was a better fit to the data than the OLS model for NP vs. SVL, NP vs. CD and NP vs. DF (Table 4).

**Table 4:** Regressions between snout-vent length (SVL), call duration (CD), number of pulses (NP) and dominant frequency (DF) in 29 *Microhyla* species based on ordinary least squares (OLS) regression and phylogenetic generalized least squares (PGLS) reg.

Models	*N*	Slope	Intercept	*r*^2^	Ln likelihood	AIC	λ	Statistical results
OLS								
CD vs. SVL	28	−1.37 ± 0.71	2.85 ± 2.13	0.126	−30.875	67.75		*F*_1, 26_ = 3.74, *P* = 0.064
DF vs. SVL	27	−1.05 ± 0.16	11.13 ± 0.48	0.633	11.640	−17.28		***F***_**1,**__**25**_ **= 43.17 *P* < 0.001**
NP vs. SVL	29	−0.40 ± 0.91	3.92 ± 2.72	0.007	−39.060	84.12		*F*_1, 27_ = 0.20, *P* = 0.660
DF vs. CD	26	0.10 ± 0.06	8.08 ± 0.10	0.089	−1.105	8.21		*F*_1,__24_ = 2.36, *P* = 0.138
NP vs. CD	28	0.65 ± 0.20	3.54 ± 0.30	0.285	−33.560	73.12		***F***_**1,**__**26**_ **= 10.34, *P* < 0.01**
NP vs. DF	27	0.95 ± 0.69	−4.81 ± 5.51	0.070	−35.430	76.86		*F*_1,__25_ = 1.89, *P* = 0.181
PGLS								
CD vs. SVL	28	−0.15 ± 0.76	−1.24 ± 2.28	0.001	−22.610	53.22	1.0	*F*_1,__26_ = 0.04, *P* = 0.845
DF vs. SVL	27	−1.09 ± 0.16	11.23 ± 0.48	0.651	14.120	−20.24	0	***F***_**1,**__**25**_ **= 46.58, *P* < 0.001**
NP vs. SVL	29	0.46 ± 0.66	1.15 ± 2.00	0.017	−19.795*	47.59	1.0	*F*_1,__27_ = 0.47, *P* = 0.497
DF vs. CD	26	0.02 ± 0.08	8.09 ± 0.20	0.004	0.640	6.72	0.777	*F*_1,__24_ = 0.09, *P* = 0.765
NP vs. CD	28	0.17 ± 0.17	2.82 ± 0.48	0.034	−19.270*	46.54	1.0	*F*_1,__26_ = 0.93, *P* = 0.344
NP vs. DF	27	0.67 ± 0.38	−2.82 ± 3.08	0.108	−17.760*	43.52	1.0	*F*_1,__25_ = 3.04, *P* = 0.094

**Note:**

* On the basis of likelihood ratio tests, the model which is labeled statistically significantly is better than the OLS regression model. Significant associations between variables are shown in bold.

## Discussion

To the best of our knowledge, the present study is the first to report the advertisement call of *M. beilunensis* consisting of 2–7 pulses per call, which has relatively few NPs among the 29 studied *Microhyla* species. Similar to that in other *Microhyla* species reported previously (Wijayathilaka, Meegaskumbura & Gianni, 2016; Sun, 2017; Nguyen et al., 2019; Poyarkov et al., 2019; Garg et al., 2018), oscillograms and spectrogram of advertisement calls in *M. beilunensis* were conformed to the pulse-repetition sound based on Beeman’s (1998) sound categories.

The patterns of variation in call parameters of anurans are correlated with female preferences and different parameters contain different kinds of biologically significant information (Gerhardt, 1991). Our results showed that the examined spectral parameters (e.g., DF, F1–F4 and CIT) were not or weakly variable as their CV_W_ is smaller than 5% and a temporal parameter (CI) was variable as its CV_W_ was larger than 10% in the three sympatric *Microhyla* species, according to the classification of Gerhardt (1991). These results were similar to those of most of reported 48 anurans, of which the DF of 69% species was classified as a static property and CI of 40% species was classified as a dynamic property (Köhler et al., 2017). Static properties with lower CV_W_ are more important in species recognition, caused by stabilizing selection, because females usually prefer the values of individual calls at or near the mean of the population. Whereas, dynamic properties with higher CV_W_ are generally supposed to be beneficial for mate choice and are driven by directional selection, because females tend to prefer extreme values of male calls (Gerhardt, 1991, 1994; Castellano & Giacoma, 1998; Wollerman, 1998; Friedl, 2006; Reinhold, 2011; Köhler et al., 2017). Therefore, our results indicated that all spectral parameters contributed to species recognition and the CI might encode information on mate quality in the three sympatric *Microhyla* species.

Generally, call parameters with stable within-individual variation have a low CV_A_ and within-individual dynamic call parameters are also more variable on the CV_A_ (Köhler et al., 2017). However, this phenomenon does not necessarily mean that among-individual variations are lower in static than in dynamic properties (Köhler et al., 2017). In our study, the CVs of spectral parameters were higher among individuals than within individual in the three *Microhyla* species. Especially, the values of CV_A_ were more than 1.8 times of those of CV_W_ for DF of the three *Microhyla* species. These variations among individuals were supposed to serve sexual selection and play a role in male–male competitions (Gerhardt, 1991; Howard & Young, 1998; Friedl & Klump, 2002; Köhler et al., 2017). Furthermore, body size is an important determinant of variations in call parameters among individuals (McClelland, Wilczynski & Ryan, 1996; Wang et al., 2012; Rodríguez et al., 2015). Our results showed that spectral parameters (e.g., DF, F1 and F3) are under the physical body size constraint, with smaller individuals producing calls at higher frequencies; in contrast, temporal parameters were not influenced by body size in the three *Microhyla* species. Similar results were also found in other *Microhyla* species (Wijayathilaka, Meegaskumbura & Gianni, 2016) indicating that spectral parameters of calls might encode the information of male’s body size in each *Microhyla* species. Therefore, researchers suggested that the spectral parameters of anurans’ advertisement calls, as an honest signal, transfer information about the body size of vocalizing individuals and thus, possibly information about its strength and quality to females and competitors (Davies & Halliday, 1978; Wells, 2007). In addition, although spectral parameters are relatively stable, they also have certain plasticity under the influence of social context (Lopez et al., 1988; Wagner, 1992; Bee & Perrill, 1996; Bee, Perrill & Owen, 2000). For example, *Lithobates clamitans* could lower the DF of their calls in response to broadcasts of conspecific calls (Bee & Perrill, 1996; Bee, Perrill & Owen, 2000). However, this phenomenon was not examined in our three *Microhyla* species. Therefore, further experiments are required to ascertain whether *Microhyla* species alter spectral parameters of advertisement calls in accordance with different social contexts.

Interspecific variation of advertisement calls in anurans serve in species recognition (Gerhardt & Huber, 2002; Köhler et al., 2017). Sympatric anuran sister species share the same acoustic space and tend to exhibit similar acoustic signals and behavioral traits (Toledo et al., 2015a). It is not clear how to minimize interference in the acoustic niche including spatial, temporal and structure dimensions (Rabin et al., 2003; Sinsch et al., 2012). The three *Microhyla* species studied used similar microhabitat and vocalizing time during the breeding season (Fei, Ye & Jiang, 2012; Z.-Q. Chen et al., 2019, unpublished data), but differences in acoustic structure were significant among the three sympatric *Microhyla* species. Besides, it cannot be ignored that phylogenetic history is perhaps the main restriction on the evolution of acoustic characteristics in anurans (Mclean, Bishop & Nakagawa, 2013). Many studies have shown that call differences between anuran species can largely be accounted by phylogeny *(Bosch & De la Riva, 2004*; Cocroft & Ryan, 1995; Mclean, Bishop & Nakagawa, 2013). Our PGLS results showed that the CD vs. SVL, NP vs. SVL, DF vs. CD, NP vs. CD and NP vs. DF relationships were significantly influenced by *Microhyla* phylogeny. However, the relationship between DF and SVL was not influenced by phylogeny, possibly owing to inter-sexual selection, which could weaken or even modify phylogenetic effects on the evolution of acoustic characteristics (Mclean, Bishop & Nakagawa, 2013). The level of call modification may also be affected by the properties of a specific signal. For example, behavioral traits are often more highly modified than physiological features (Blomberg, Garland & Ives, 2003; Mclean, Bishop & Nakagawa, 2013). Several *Microhyla* species are sympatric (Lee et al., 2016; Poyarkov et al., 2019; Frost, 2020; Z.-Q. Chen et al., 2019, unpublished data). To prevent inter-specific hybridization events, sympatric species must be able to distinguish conspecifics, via behavioral and physical features. Gerhardt & Huber (2002) found that inter-sexual selection is the main driving force behind the evolution of acoustic communication in anurans. Our results provide considerable evidence that the three sympatric *Microhyla* species have acoustic divergence in call structure, facilitating inter-sexual selection, which may therefore help prevent hybridization.

## Conclusion

Based on the results, we conclude the following: (1) CVs of all spectral parameters were smaller than 5%, whereas the CV of CI was larger than 10% within individual of the three sympatric *Microhyla* species. (2) Body size was a key factor that leads to among-individual variation in advertisement calls of *Microhyla* species. (3) Acoustic divergence in call structure existed in the three sympatric *Microhyla* species. (4) The PGLS analysis showed that phylogeny affected the NP, DF and SVL vs. CD and SVL and DF vs. NP but not DF vs. SVL.

## Supplemental Information

10.7717/peerj.8708/supp-1 Supplemental Information 1 Descriptive statistics for mean and range of snout-vent length (SVL), call duration (CD), number of pulses (NP) and dominant frequency (DF) of advertisement calls of 29 *Microhyla* species. Data were collected from 21 references and our study.

10.7717/peerj.8708/supp-2 Supplemental Information 2 Vouchers, localities and GenBank accession numbers for samples used in the molecular analyses.

10.7717/peerj.8708/supp-3 Supplemental Information 3 The Bayesian inference tree for 29 *Microhyla* species reconstructed based on mitochondrial 12S+16S+COⅠ gene sequences (1,436 bp). One *Kaloula verrucosa* sample is chosen as outgroup. Numbers on the nodes show posterior probabilities. The scaleplate represents substitutions/site. The specimen number and accession number are showed in appendix table 2.

10.7717/peerj.8708/supp-4 Supplemental Information 4 Raw data of all advertisement calls in the three *Microhyla* species.

10.7717/peerj.8708/supp-5 Supplemental Information 5 Raw data of individual calls in the three *Microhyla* species.

10.7717/peerj.8708/supp-6 Supplemental Information 6 DATA of Microhyla 12S+16S+COI.

10.7717/peerj.8708/supp-7 Supplemental Information 7 Audio file of advertisement calls in Microhyla beilunensis (Voucher: MB001) from Zhejiang, East China.

10.7717/peerj.8708/supp-8 Supplemental Information 8 Audio file of advertisement calls in Microhyla beilunensis (Voucher: MB002) from Zhejiang, East China.

10.7717/peerj.8708/supp-9 Supplemental Information 9 Audio file of advertisement calls in Microhyla beilunensis (Voucher: MB002) from Zhejiang, East China.

10.7717/peerj.8708/supp-10 Supplemental Information 10 Audio file of advertisement calls in Microhyla beilunensis (Voucher: MB013) from Zhejiang, East China.

10.7717/peerj.8708/supp-11 Supplemental Information 11 Audio file of advertisement calls in Microhyla beilunensis (Voucher: MB017) from Zhejiang, East China.

10.7717/peerj.8708/supp-12 Supplemental Information 12 Audio file of advertisement calls in Microhyla beilunensis (Voucher: MB019) from Zhejiang, East China.

10.7717/peerj.8708/supp-13 Supplemental Information 13 Audio file of advertisement calls in Microhyla beilunensis (Voucher: MB022) from Zhejiang, East China.

10.7717/peerj.8708/supp-14 Supplemental Information 14 Audio file of advertisement calls in Microhyla beilunensis (Voucher: MB025) from Zhejiang, East China.

10.7717/peerj.8708/supp-15 Supplemental Information 15 Audio file of advertisement calls in Microhyla beilunensis (Voucher: MB026) from Zhejiang, East China.

10.7717/peerj.8708/supp-16 Supplemental Information 16 Audio file of advertisement calls in Microhyla beilunensis (Voucher: MB028) from Zhejiang, East China.

10.7717/peerj.8708/supp-17 Supplemental Information 17 Audio file of advertisement calls in Microhyla beilunensis (Voucher: MB0301) from Zhejiang, East China.

10.7717/peerj.8708/supp-18 Supplemental Information 18 Audio file of advertisement calls in Microhyla beilunensis (Voucher: MB0302) from Zhejiang, East China.

10.7717/peerj.8708/supp-19 Supplemental Information 19 Audio file of advertisement calls in Microhyla beilunensis (Voucher: MB0351) from Zhejiang, East China.

10.7717/peerj.8708/supp-20 Supplemental Information 20 Audio file of advertisement calls in Microhyla beilunensis (Voucher: MB0352) from Zhejiang, East China.

10.7717/peerj.8708/supp-21 Supplemental Information 21 Audio file of advertisement calls in Microhyla beilunensis (Voucher: MB0361) from Zhejiang, East China.

10.7717/peerj.8708/supp-22 Supplemental Information 22 Audio file of advertisement calls in Microhyla beilunensis (Voucher: MB03612) from Zhejiang, East China.

10.7717/peerj.8708/supp-23 Supplemental Information 23 Audio file of advertisement calls in Microhyla beilunensis (Voucher: MB0362) from Zhejiang, East China.

10.7717/peerj.8708/supp-24 Supplemental Information 24 Audio file of advertisement calls in *Microhyla fissipes* (Voucher: MF170501006001) from Zhejiang, East China.

10.7717/peerj.8708/supp-25 Supplemental Information 25 Audio file of advertisement calls in *Microhyla fissipes* (Voucher: MF170501006002) from Zhejiang, East China.

10.7717/peerj.8708/supp-26 Supplemental Information 26 Audio file of advertisement calls in *Microhyla fissipes* (Voucher: MF170501006003) from Zhejiang, East China.

10.7717/peerj.8708/supp-27 Supplemental Information 27 Audio file of advertisement calls in *Microhyla fissipes* (Voucher: MF180421001) from Zhejiang, East China.

10.7717/peerj.8708/supp-28 Supplemental Information 28 Audio file of advertisement calls in *Microhyla fissipes* (Voucher: MF180421002) from Zhejiang, East China.

10.7717/peerj.8708/supp-29 Supplemental Information 29 Audio file of advertisement calls in *Microhyla fissipes* (Voucher: MF180421005) from Zhejiang, East China.

10.7717/peerj.8708/supp-30 Supplemental Information 30 Audio file of advertisement calls in *Microhyla fissipes* (Voucher: MF180421007) from Zhejiang, East China.

10.7717/peerj.8708/supp-31 Supplemental Information 31 Audio file of advertisement calls in *Microhyla fissipes* (Voucher: MF180421009) from Zhejiang, East China.

10.7717/peerj.8708/supp-32 Supplemental Information 32 Audio file of advertisement calls in *Microhyla fissipes* (Voucher: MF180421013) from Zhejiang, East China.

10.7717/peerj.8708/supp-33 Supplemental Information 33 Audio file of advertisement calls in *Microhyla fissipes* (Voucher: MF180421021) from Zhejiang, East China.

10.7717/peerj.8708/supp-34 Supplemental Information 34 Audio file of advertisement calls in *Microhyla fissipes* (Voucher: MF170501006019) from Zhejiang, East China.

10.7717/peerj.8708/supp-35 Supplemental Information 35 Audio file of advertisement calls in *Microhyla fissipes* (Voucher: MF170501006024) from Zhejiang, East China.

10.7717/peerj.8708/supp-36 Supplemental Information 36 Audio file of advertisement calls in *Microhyla fissipes* (Voucher: MF170501006028) from Zhejiang, East China.

10.7717/peerj.8708/supp-37 Supplemental Information 37 Audio file of advertisement calls in *Microhyla heymonsi* (Voucher: MH170501004001) from Zhejiang, East China.

10.7717/peerj.8708/supp-38 Supplemental Information 38 Audio file of advertisement calls in *Microhyla heymonsi* (Voucher: MH170501004002) from Zhejiang, East China.

10.7717/peerj.8708/supp-39 Supplemental Information 39 Audio file of advertisement calls in *Microhyla heymonsi* (Voucher: MH170501004003) from Zhejiang, East China.

10.7717/peerj.8708/supp-40 Supplemental Information 40 Audio file of advertisement calls in *Microhyla heymonsi* (Voucher: MH170501004004) from Zhejiang, East China.

10.7717/peerj.8708/supp-41 Supplemental Information 41 Audio file of advertisement calls in *Microhyla heymonsi* (Voucher: MH170501004005) from Zhejiang, East China.

10.7717/peerj.8708/supp-42 Supplemental Information 42 Audio file of advertisement calls in *Microhyla heymonsi* (Voucher: MH170501004006) from Zhejiang, East China.

10.7717/peerj.8708/supp-43 Supplemental Information 43 Audio file of advertisement calls in *Microhyla heymonsi* (Voucher: MH170501004008) from Zhejiang, East China.

10.7717/peerj.8708/supp-44 Supplemental Information 44 Audio file of advertisement calls in *Microhyla heymonsi* (Voucher: MH170501004009) from Zhejiang, East China.

10.7717/peerj.8708/supp-45 Supplemental Information 45 Audio file of advertisement calls in *Microhyla heymonsi* (Voucher: MH170501004010) from Zhejiang, East China.

10.7717/peerj.8708/supp-46 Supplemental Information 46 Audio file of advertisement calls in *Microhyla heymonsi* (Voucher: MH170501004011) from Zhejiang, East China.

10.7717/peerj.8708/supp-47 Supplemental Information 47 Audio file of advertisement calls in *Microhyla heymonsi* (Voucher: MH170501004012) from Zhejiang, East China.

10.7717/peerj.8708/supp-48 Supplemental Information 48 Audio file of advertisement calls in *Microhyla heymonsi* (Voucher: MH170501004013) from Zhejiang, East China.

10.7717/peerj.8708/supp-49 Supplemental Information 49 Audio file of advertisement calls in *Microhyla heymonsi* (Voucher: MH170501004014) from Zhejiang, East China.

10.7717/peerj.8708/supp-50 Supplemental Information 50 Audio file of advertisement calls in *Microhyla heymonsi* (Voucher: MH170502002018) from Zhejiang, East China.

10.7717/peerj.8708/supp-51 Supplemental Information 51 Audio file of advertisement calls in *Microhyla heymonsi* (Voucher: MH170502002019) from Zhejiang, East China.

## Additional Information and Declarations

### Field Study Permissions

The following information was supplied relating to field study approvals (i.e., approving body and any reference numbers): Administration Bureau of Jiulongshan National Nature Reserve provided the permit for capturing animals in the field (20180422).

### Data Availability

The following information was supplied regarding data availability: Raw data of advertisement calls of three species and recording files are available as Supplemental Files.

## References

[ref-1] AmphibiaChina (2020). The database of Chinese amphibians. Kunming Institute of Zoology (CAS), Kunming, Yunnan, China. http://www.amphibiachina.org/.

[ref-2] Barros FC, Herrel A, Kohlsdorf T (2011). Head shape evolution in Gymnophthalmidae: does habitat use constrain the evolution of cranial design in fossorial lizards?. Journal of Evolutionary Biology.

[ref-3] Bee MA, Perrill SA (1996). Responses to conspecific advertisement calls in the green frog (*Rana clamitans*) and their role in male–male communication. Behaviour.

[ref-4] Bee MA, Perrill SA, Owen PC (2000). Male green frogs lower the pitch of acoustic signals in defense of territories: a possible dishonest signal of size?. Behavioral Ecology.

[ref-5] Bee MA, Reichert MS, Tumulty JP (2016). Assessment and recognition of rivals in anuran contests. Advances in the Study of Behavior.

[ref-6] Bee MA, Suyesh R, Biju SD (2013). Vocal behavior of the Ponmudi Bush Frog (*Raorchestes graminirupes*): repertoire and individual variation. Herpetologica.

[ref-7] Beeman K, Hopp SL, Owren MJ, Evans CS (1998). Digital signal analysis, editing, and synthesis. Animal Acoustic Communication: Sound Analysis and Research Methods.

[ref-8] Blomberg SP, Garland T, Ives AR (2003). Testing for phylogenetic signal in comparative data: behavioural traits are more labile. Evolution.

[ref-9] Boersma P, Weenink D (2019). http://www.praat.org/.

[ref-10] Bosch J, De la Riva I (2004). Are frog calls modulated by the environment? An analysis with anuran species from Bolivia. Canadian Journal of Zoology.

[ref-11] Boulenger GA (1884). Descriptions of new species of reptiles and batrachians in the British Museum.—Part. II. Annals and Magazine of Natural History.

[ref-12] Castellano S, Giacoma C (1998). Stabilizing and directional female choice for male calls in the European green toad. Animal Behaviour.

[ref-13] Castellano S, Rosso A (2006). Variation in call temporal properties and female preferences in *Hyla intermedia*. Behaviour.

[ref-14] Cocroft RB, Ryan MJ (1995). Patterns of advertisement call evolution in toads and chorus frogs. Animal Behaviour.

[ref-15] Darriba D, Taboada GL, Ramón D, Posada D (2012). Jmodeltest 2: more models, new heuristics and parallel computing. Nature Methods.

[ref-16] Davies NB, Halliday TR (1978). Deep croaks and fighting assessment in toads, *Bufo bufo*. Nature.

[ref-17] Fei L, Ye CY, Jiang JP (2012). Colored Atlas of Chinese Amphibians and their distributions.

[ref-18] Friedl TWP (2006). Individual male calling pattern and male mating success in the European treefrog (*Hyla arborea*): is there evidence for directional or stabilizing selection on male calling behaviour?. Ethology.

[ref-19] Friedl TWP, Klump GM (2002). The vocal behaviour of male European treefrogs (*Hyla arborea*): implications for inter- and intrasexual selection. Behaviour.

[ref-20] Frost DR (2020). http://research.amnh.org/herpetology/amphibia/index.php/.

[ref-21] Gambale PG, Signorelli L, Bastos RP (2014). Individual variation in the advertisement calls of a Neotropical treefrog (*Scinax constrictus*). Amphibia-Reptilia.

[ref-22] Garg S, Suyesh R, Das A, Jiang JP, Wijayathilaka N, Amarasinghe AAT, Alhadi F, Vineeth KK, Aravind NA, Senevirathne G, Meegaskumbura M, Biju SD (2018). Systematic revision of *Microhyla* (Microhylidae) frogs of South Asia: a molecular, morphological, and acoustic assessment. Vertebrate Zoology.

[ref-23] Gasser H, Amézquita A, Hödl W (2009). Who is calling? Intraspecific call variation in the aromobatid frog *Allobates femoralis*. Ethology.

[ref-24] Gerhardt HC (1991). Female mate choice in treefrogs: static and dynamic acoustic criteria. Animal Behaviour.

[ref-25] Gerhardt HC (1994). Reproductive character displacement of female mate choice in the grey treefrog, *Hyla chrysoscelis*. Animal Behaviour.

[ref-26] Gerhardt HC, Huber F (2002). Acoustic communication in insects and anurans: common problems and diverse solutions.

[ref-27] Gingras B, Boeckle M, Herbst CT, Fitch WT (2013). Call acoustics reflect body size across four clades of anurans. Journal of Zoology.

[ref-28] Harrell FE (2012). http://CRAN.R-project.org/package=rms.

[ref-29] Hoffmann F, Kloas W (2012). Estrogens can disrupt amphibian mating behavior. PLOS ONE.

[ref-30] Howard RD, Young JR (1998). Individual variation in male vocal traits and female mating preferences in *Bufo americanus*. Animal Behaviour.

[ref-31] Hurvich CM, Tsai CL (1989). Regression and time series model selection in small samples. Biometrika.

[ref-32] Kelley DB (2004). Vocal communication in frogs. Current Opinion in Neurobiology.

[ref-33] Köhler J, Jansen M, Rodríguez A, Kok PJR, Toledo LF, Emmrich M, Glaw F, Haddad CFB, Rödel MO, Vences M (2017). The use of bioacoustics in anuran taxonomy: theory, terminology, methods and recommendations for best practice. Zootaxa.

[ref-34] Lee K-H, Shaner P-JL, Lin Y-P, Lin S-M (2016). Geographic variation in advertisement calls of a *Microhylid* frog-testing the role of drift and ecology. Ecology and Evolution.

[ref-35] Lingnau R, Bastos RP (2007). Vocalizations of the Brazilian torrent frog *Hylodes heyeri* (Anura: Hylodidae): repertoire and influence of air temperature on advertisement call variation. Journal of Natural History.

[ref-36] Lopez PT, Narins PM, Lewis ED, Moore SW (1988). Acoustically induced call modification in the white-lipped frog, *Leptodactylus albilabris*. Animal Behaviour.

[ref-37] Matsui M, Hamidy A, Belabut DM, Ahmad N, Panha S, Sudin A, Khonsue W, Oh HS, Yong HS, Jiang JP (2011). Systematic relationships of oriental tiny frogs of the family Microhylidae (Amphibia, Anura) as revealed by mtDNA genealogy. Molecular Phylogenetics & Evolution.

[ref-38] McClelland BE, Wilczynski W, Ryan MJ (1996). Correlations between call characteristics and morphology in male cricket frogs (*Acris crepitans*). Journal of Experimental Biology.

[ref-39] Mclean MJ, Bishop PJ, Nakagawa S (2013). Assessing the patterns of evolution in anuran vocal sexual signals. Evolutionary Biology.

[ref-40] Morais AR, Batista VG, Gambale PG, Signorelli L, Bastos RP (2012). Acoustic communication in a Neotropical frog (*Dendropsophus minutus*): vocal repertoire, variability and individual discrimination. Herpetological Journal.

[ref-41] Nguyen LT, Poyarkov NA, Nguyen TT, Nguyen TA, Nguyen VH, Gorin VA, Murphy RW, Nguyen SN (2019). A new species of the genus *Microhyla* Tschudi, 1838 (Amphibia: Anura: Microhylidae) from Tay Nguyen Plateau. Central Vietnam. Zootaxa.

[ref-42] Orme D, Freckleton R, Thomas G, Petzoldt T, Fritz S, Isaac N (2012). http://CRAN.R-project.org/package=caper.

[ref-43] Pettit BA, Bourne GR, Bee MA (2013). Advertisement call variation in the golden rocket frog (*Anomaloglossus beebei*): evidence for individual distinctiveness. Ethology.

[ref-44] Poyarkov Jr, Nikolay A, Zaw T, Kretova D, Gogoleva S, Pawangkhanant P, Che J (2019). On the road to Mandalay: contribution to the *Microhyla* Tschudi, 1838 (Amphibia: Anura: Microhylidae) fauna of Myanmar with description of two new species. Zoological Research.

[ref-45] Pröhl H, Eulenberg J, Meuche I, Bolaños F (2013). Parasite infection has little effect on sexual signals and reproductive behaviour in strawberry poison frogs. Evolutionary Ecology.

[ref-46] Pröhl H, Hagemann S, Karsh J, Höbel G (2007). Geographic variation in male sexual signals in strawberry poison frogs (*Dendrobates pumilio*). Ethology.

[ref-47] R Development Core Team (2015). R: a language and environment for statistical computing.

[ref-48] Rabin LA, Mccowan B, Hooper SL, Owings DH (2003). Anthropogenic noise and its effect on animal communication: an interface between comparative psychology and conservation biology. International Journal of Comparative Psychology.

[ref-49] Reichert MS, Gerhardt HC (2013). Gray tree frogs, *Hyla versicolor*, give lower-frequency aggressive calls in more escalated contests. Behavioral Ecology and Sociobiology.

[ref-50] Reinhold K (2009). Variation of acoustic courtship signals in insects and amphibians: no evidence for bimodality, but identical dependence on duration. Ethology.

[ref-51] Reinhold K (2011). Variation in acoustic signalling traits exhibits footprints of sexual selection. Evolution.

[ref-52] Rodríguez RL, Araya-Salas M, Gray DA, Reichert MS, Symes LB, Wilkins MR, Safran RJ, Höbel G (2015). How acoustic signals scale with individual body size: common trends across diverse taxa. Behavioral Ecology.

[ref-53] Ronquist F, Teslenko M, Van Der Mark P, Ayres DL, Darling A, Höhna S, Larget B, Liu L, Suchard MA, Huelsenbeck JP (2012). MrBayes 3.2: efficient Bayesian phylogenetic inference and model choice across a large model space. Systematic Biology.

[ref-54] Sinsch U, Lümkemann K, Rosar K, Schwarz C, Dehling M (2012). Acoustic niche partitioning in an anuran community inhabiting an *Afromontane wetland* (Butare, Rwanda). African Zoology.

[ref-55] Sun ZX (2017). A comparison of acoustic structure of vocalization in different habitat frog species in the Mt. Diaoluo National Nature Reserve.

[ref-56] Toledo LF, Llusia D, Vieira CA, Corbo M, Márquez R (2015a). Neither convergence nor divergence in the advertisement call of sympatric congeneric Neotropical treefrogs. Bioacoustics.

[ref-57] Toledo LF, Martins IA, Bruschi DP, Passos MA, Alexandre C, Haddad CFB (2015b). The anuran calling repertoire in the light of social context. Acta Ethologica.

[ref-58] Turin RAF, Nali RC, Prado CPA (2018). Intraspecific call variation in a Neotropical gladiator frog with a complex advertisement call. Amphibia-Reptilia.

[ref-59] Vogt T (1911). Beitrag zur Amphibien-fauna der Insel Formosa.

[ref-60] Wagner WE (1992). Deceptive or honest signalling of fighting ability? A test of alternative hypotheses for the function of changes in call dominant frequency by male cricket frogs. Animal Behaviour.

[ref-61] Wang J, Cui J, Shi H, Brauth SE, Tang Y (2012). Effects of body size and environmental factors on the acoustic structure and temporal rhythm of calls in Rhacophorus dennysi. Asian Herpetological Research.

[ref-62] Warne RW, Charnov EL (2008). Reproductive allometry and the size-number trade-off for lizards. American Naturalist.

[ref-63] Wei L, Shao WW, Fan XL, Flanders J, Ding GH, Lin ZH (2019). Advertisement calls of Guenther’s frog *Hylarana guentheri* (Anura: Ranidae) during the breeding season. Bioacoustics.

[ref-64] Wells KD (2007). The ecology and behavior of amphibians.

[ref-65] Wijayathilaka N, Meegaskumbura M, Gianni P (2016). An acoustic analysis of the Genus *Microhyla* (Anura: Microhylidae) of Sri Lanka. PLOS ONE.

[ref-66] Wollerman L (1998). Stabilizing and directional preferences of female *Hyla ebraccata* for calls differing in static properties. Animal Behaviour.

[ref-67] Yu DU, Longhui LIN, Yuntao YAO, Chixian LIN, Xiang JI (2014). Body size and reproductive tactics in varanid lizards. Asian Herpetological Research.

[ref-68] Zhang MH, Fei L, Ye CY, Wang WF, Wang B, Jiang JP (2018). A new species of genus *Microhyla* (Amphibia: Anura: Microhylidae) from Zhejiang Province. China Asian Herpetological Research.

[ref-69] Ziegler L, Arim M, Bozinovic F (2015). Intraspecific scaling in frog calls: the interplay of temperature, body size and metabolic condition. Oecologia.

