# Supplementary material for: Acoustic divergence in advertisement calls among three sympatric Microhyla species from East China
Source: PeerJ. 2020 Mar 11;8:e8708. doi: 10.7717/peerj.8708 (PMC7071819; doi:10.7717/peerj.8708)
Supplement: Supplemental Information 1 — Data were collected from 21 references and our study. [file peerj-08-8708-s001.doc]

**Table S1 Descriptive statistics for** **mean and range of snout-vent length (SVL), call duration (CD), number of pulses (NP) and dominant frequency (DF) of advertisement calls of 29 *Microhyla* species.** Data were collected from 21 references and our study.

| Species | SVL (mm) | CD (s) | NP | DF (Hz) | References |
| --- | --- | --- | --- | --- | --- |
| *Microhyla achatina* | 21.6  (21.1-22.3) | 0.306  (0.116-0.508) | 8  (4-11) | 3173.0  (2718-3400) | *Arini et al., 2016; Garg et al., 2019; Poyarkov et al., 2019* |
| *Microhyla aurantiventris* | 26.2  (25.2-27.0) | 0.154  (0.125-0.227) | 20.2  (15-26) | 2100.0  (1800-2200) | *Nguyen, 2019* |
| *Microhyla beilunensis* | 22.5 | 0.273  (0.248-0.331) | 5.0  (2-7) | 2139.2  (1750.1-2411.7) | *our study* |
| *Microhyla berdmorei* | 26.5 | 0.175  (0.09-0.26) | 6  (3–9) | 1650.0  (1500-1800) | *Heyer, 1971* |
| *Microhyla borneensis* | 19.0  (17-21) | 0.169  (0.104-0.242) | 5.7  (2-9) | 2404.0  (2250–2530) | *Dehling, 2010; Poyarkov et al., 2019* |
| *Microhyla butleri* | 20.8  (20.6-21.0) | 0.259  (0.16-0.354) | 36.4  (34-40) | 2562.9  (1200-4500) | *Sun, 2017; Nguyen, 2019* |
| *Microhyla darreli* | 15.3  (15.0-15.7) | 0.654  (0.5903-0.7362) | 68  (63-78) | 3600 | *Garg et al., 2019* |
| *Microhyla fanjingshanensis* | 21.4  (19.0-22.7) | 0.493  (0.307-0.660) | 10.7  (10-12) | 2337.3  (2306.9-2375.7) | *Li et al., 2019* |
| *Microhyla fissipes* | 22.3  (22.1-23.2) | 0.264  (0.199-0.358) | 14.9  (11.3-16.6) | 2773.5  (1520-3100) | *Zhou et al., 2014; Wei et al., 2013; Garg et al., 2019; our study* |
| *Microhyla heymonsi* | 19.5  (16-24.4) | 0.320  (0.171-0.48) | 6.3  (5.3-11) | 3033.5  (1510-4700) | *Heyer, 1971; Grosselet et al., 2004; Garcia-Rutledge & Narins, 2001; Poyarkov et al., 2019; our studyy* |
| *Microhyla irrawaddy* | 14.9  (12.3-17.1) | 0.180  (0.004–0.28) | 4  (1-5) | – | *Poyarkov et al., 2019* |
| *Microhyla karunaratnei* | 15.7  (13.8-16.7) | 0.868  (0.699-1.172) | 63.3  (50–95) | 3250.0  (3100-3400) | *Wijayathilaka & Meegaskumbura, 2016; Garg et al., 2019* |
| *Microhyla kodial* | 17.2  (16.9-17.4) | 0.330  (0.11–0.42) | 6  (2-7) | 3752.2  (3359.22-4220.5) | *Vineeth et al., 2018; Garg et al., 2019* |
| *Microhyla laterite* | 15.7  (14.3-16.6) | 0.765  (0.60-0.89) | 97  (79–113) | 5029.0  (3500-6607) | *Seshadri et al., 2016; Garg et al., 2019* |
| *Microhyla malang* | 20.5  (18.7-22.2) | 0.169  (0.104−0.242) | 6  (4–8) | 2404.0  (2250−2530) | *Dehling, 2010; Matsui, 2011* |
| *Microhyla marmorata* | 20.2  (18.8-21.5)* | – | 11  (9–13) | 2885.5  (2756-3015) | *Dung et al., 2016* |
| *Microhyla mihintalei* | 24.6 | 0.187  (0.141–0.245) | 12.5  (9–15) | 2100.0  (1300-2600) | *Wijayathilaka & Meegaskumbura, 2016* |
| *Microhyla mukhlesuri* | 18.8  (16.5-21.0)* | 0.270 | 14  (10–18) | – | *Heyer, 1971* |
| *Microhyla mymensinghensis* | 21.9  (20.5-23.9) | 0.466  (0.4502-0.4771) | 21  (19-22) | 3600.0  (3500-3600) | *Garg et al., 2019* |
| *Microhyla nilphamariensis* | 21.7  (19.3-23.8) | 0.337  (0.3113-0.3687) | 11  (10-12) | 2300.0 | *Garg et al., 2019* |
| *Microhyla orientalis* | 17.0  (16.2-18.3) | 0.062  (0.482-0.674) | 5  (3-5) | 3700.0 | *Garg et al., 2019; Poyarkov et al., 2019* |
| *Microhyla ornata* | 21.4  (19.2-24.9) | 0.285  (0.21-0.795) | 11.9  (9-14) | 3228.2  (1000-4000) | *Kuramoto & Joshy, 2006; Wijayathilaka & Meegaskumbura, 2016; Vineeth et al., 2018; Garg et al., 2019* |
| *Microhyla palmipes* | 16.0 | 0.110  (0.058.2-0.1644) | 11  (6-13) | 3500.0  (3400-3500) | *Bain& Nguyen, 2004; Garg et al., 2019* |
| *Microhyla petrigena* | 15.0  (14-16) | 0.133  (0.069-0.174) | 12.1  (6–17) | 4430.0  (3850-5050) | *Dehling, 2010* |
| *Microhyla pulchra* | 33.6 | 0.063 | 59  (38–80) | 2074.0 | *Xu et al., 2005; Dung et al., 2016 Sun, 2017* |
| *Microhyla rubra* | 27.6  (24.8-29.6) | 0.156  (0.115-0.228) | 14.5  (10-21) | 2234.0  (2000-2268) | *Kanamadi et al., 1994; Garg et al., 2019* |
| *Microhyla sholigari* | 16.6  (16.1-18.0) | 0.826  (0.53-1.012) | 67.5  (56-79) | 3560.2  (3400-3779) | *Seshadri et al., 2016; Vineeth et al., 2018; Garg et al., 2019* |
| *Microhyla taraiensis* | 20.46 | 0.750  (0.688-0.911) | 13.5  (13–14) | 3305.5  (3433 3101) | *Khatiwada et al., 2017* |
| *Microhyla zeylanica* | 18.2  (17.3-19.3) | 1.806  (1.503-1.999) | 85  (61-92) | 2650.0  (2200-3000) | *Wijayathilaka & Meegaskumbura, 2016; Garg et al., 2019* |

^*^ data from **Poyarkov NA, Vassilieva AB, Orlov NL, Galoyan EA, Dao TTA, Le DTT, Kretova VD, Peter Geissler P. 2014.** Taxonomy and distribution of narrow-mouth frogs of the genus *Microhyla* Tschudi, 1838 (Anura: Microhylidae) from Vietnam with descriptions of five new species. *Russian Journal of Herpetology* 21:89-148

**References**

**Arini K, Noer MI, Wulandari A, Amalia R, Auliandina T. 2016.** Temporal and spectral variation in advertisement call of males *Microhyla achatina* (Tschudi, 1838) are sufficient for individual discrimination. *AIP Conference Proceedings*, **1744**:020032 DOI 10.1063/1.4953506.

**Bain RH, Nguyen QT.** 2004. Three new species of narrow-mouth frogs (Genus: *Microhyla*) from Indochina, with comments on Microhyla annamensis and Microhyla palmipes. *Copeia*, **2004**:507−524 DOI 10.2307/1448467.

**Dehling JM. 2010.** Advertisement calls of two species of *Microhyla* (Anura: Microhylidae) from Borneo. *Salamandra*, **46**:114−116.

**Garcia-Rutledge EJ, Narins PM.** 2001. Shared acoustic resources in an old world frog community. *Herpetologica*, **57**:104−116 DOI 10.2307/3893144.

**Garg S, Suyesh R, Das A, Jiang JP, Wijayathilaka N, Amarasinghe AAT, Alhadi F, Vineeth KK, Aravind NA, Senevirathne G, Meegaskumbura M, Biju SD.** 2018. Systematic revision of *Microhyla* (Microhylidae) frogs of South Asia: a molecular, morphological, and acoustic assessment. *Vertebrate Zoology*, **69**:1−71 DOI 10.26049/VZ69-1-2019-01.

**Grosselet O, Sengupta S, Gupta A, Vauche M, Gupta S. 2004.** *Microhyla heymonsi* Vogt, 1911 (Anura: Microhylidae) from mainland India, with bioacoustic analysis of its advertising call. *Hamadryad*, **29**:131-133.

**Heyer WR. 1971.** Mating calls of some frogs from Thailand. *Fieldiana Zoology*, **58**:61-82.

**Kanamadi R, Hiremath C, Schneider H. 1994.** Courtship, amplexus and advertisement call of the frog, *Microhyla rubra*. *Current Science*, **66**:683−684.

**Kuramoto M, Joshy SH. 2006.** Morphological and acoustic comparisons of *Microhyla ornata, M. fissipes*, and *M. okinavensis* (Anura: Microhylidae). *Current Herpetology*, **25**:15-27 DOI 10.3105/1345-5834(2006)25[15:MAACOM]2.0.CO;2.

**Dung LT, Hoa NT, Anh LM, Truong NQ.** 2016. Advertisement call and description of the tadpole of *Microhyla* marmorata Bain & Nguyen, 2004 from Xuan Son National Park, Phu Tho Province. *Vietnam Journal of Biology*, **38**:154−164 DOI 10.15625/0866-7160/v38n2.7738.

**Li S, Zhang M, Xu N, Lv JC, Jiang JP, Liu J, Wei G, Wang B.** 2019. A new species of the genus *Microhyla* (Amphibia: Anura: Microhylidae) from Guizhou Province, China. *Zootaxa*, **4624**:551−575 DOI 10.11646/zootaxa.4624.4.7.

**Matsui M.** 2011. Taxonomic revision of one of the Old World's smallest frogs, with description of a new bornean *Microhyla* (Amphibia, Microhylidae). *Zootaxa*, **2814**:33−49 DOI 10.11646/zootaxa.2814.1.3.

**Nguyen LT, Poyarkov NA Jr, Nguyen TT, Nguyen TA, Nguyen VH, Gorin VA, Murphy RW, Nguyen SN.** 2019. A new species of the genus *Microhyla* Tschudi, 1838 (Amphibia: Anura: Microhylidae) from Tay Nguyen Plateau, Central Vietnam. *Zootaxa*, **4543**:549−580 DOI 10.11646/zootaxa.4543.4.4.

**Poyarkov Jr, Nikolay A., Zaw T, Kretova D, Gogoleva S, Pawangkhanant P, Che J. 2019.** On the road to Mandalay: contribution to the *Microhyla* Tschudi, 1838 (Amphibia: Anura: Microhylidae) fauna of Myanmar with description of two new species. *Zoological Research*, **40**:244-276 DOI 10.24272/j.issn.2095-8137.2019.044.

**Seshadri KS, Singal R, Priti H, Ravikanth G, Vidisha MK, Saurabh S, Pratik M, Gururaja KV. 2016.** *Microhyla laterite* sp. nov., A New Species of *Microhyla* Tschudi, 1838 (Amphibia: Anura: Microhylidae) from a Laterite Rock Formation in South West India. *PLoS ONE*, **11**:e0149727 DOI 10.1371/journal.pone.0149727.

**Sun ZX. 2017.** A comparison of acoustic structure of vocalization in different habitat frog species in the Mt. Diaoluo National Nature Reserve. Haikou: Hainan Normal University.

**Vineeth KK, Radhakrishna U, Godwin R., Anwesha S, Rajashekhar KP, Aravind N.** 2018. A new species of *Microhyla* Tschudi, 1838 (Anura: Microhylidae) from West Coast of India: an integrative taxonomic approach. *Zootaxa*, **4420**:151–179 DOI 10.11646/zootaxa.4420.2.1.

**Wei L, Shao WW, Lin ZH. 2013.** Characteristics of courtship calls of *Microhyla ornata* (Anura: Microhylidae). *Zoological Research*, **34**:14-20 DOI 10.3724/SP.J.1141.2013.01014.

**Wijayathilaka N, Meegaskumbura M. 2016.** An Acoustic Analysis of the Genus *Microhyla* (Anura: Microhylidae) of Sri Lanka. *PLoS ONE*, **11**:e0159003 DOI 10.1371/journal.pone.0159003.

**Xu XF, Xie F, Jiang JP, Mo YM, Zheng ZH. 2005.** The acoustic features of the mating call of 12 anuran species. *Chinese Journal of Zoology*, **40**:12-19 DOI 10.1360/jos162021.

**Zhou YL, Qiu X, Fang XB, Yang LY, Zhao Y, Fang T, Zheng WH, Liu JS. 2014.** Acoustic characteristics of eight common Chinese anurans during the breeding season. *Zoological Research*,**35**:42-50 DOI 10.11813/j.issn.0254-5853.2014.1.042.
