# Supplementary material for: Acoustic divergence in advertisement calls among three sympatric Microhyla species from East China
Source: PeerJ. 2020 Mar 11;8:e8708. doi: 10.7717/peerj.8708 (PMC7071819; doi:10.7717/peerj.8708)
Supplement: Supplemental Information 2 [file peerj-08-8708-s002.doc]

**Table S2 Vouchers, localities and GenBank accession numbers for samples used in the molecular analyses.**

| ID | Species | Voucher | Locality | GenBank Accession number | | |
| --- | --- | --- | --- | --- | --- | --- |
|  |  |  |  | 12S | 16S | COI |
| 1 | *Microhyla achatina* | PK8906_(BiologiUGM) | Pekalongan, Jawa Tengah, Jawa, Indonesia | MK034338 | MK034338 | – |
| 2 | *Microhyla aurantiventris* | ITBCZ 4360 | Gia Lai, Tram Lap,Vietnam | – | MH286426 | – |
| 3 | *Microhyla beilunensis* | CIBBL002 | Chaiqiao, Beilun, Ningbo, Zhejiang, China | MH234521 | MH234535 | MH234543 |
| 4 | *Microhyla berdmorei* | KUHE:20654 | Phrae, Thailand | LC465690 | LC465690 | – |
| 5 | *Microhyla borneensis* | KUHE:53938 | Serapi, Sarawak, Malaysia | AB634605 | AB634663 | – |
| 6 | *Microhyla butleri* | MB1 | China | KT285802 | KT285802 | KT285802 |
| 7 | *Microhyla darreli* | ZSI/WGRC/V/A/962 | Thiruvananthapuram, Karamana, India | – | MH807390 | – |
| 8 | *Microhyla fanjingshanensis* | CIBFJS20180425006 | Fanjing Mountain, Guizhou, China | MK087853 | MK087856 | MK087859 |
| 9 | *Microhyla fissipes* | – | Taiwan, China | MF673131 | MF673131 | MF673131 |
| 10 | *Microhyla heymonsi* | – | – | AY458596 | AY458596 | AY458596 |
| 11 | *Microhyla irrawaddy* | ZMMU:A5976 | Myanmar | MK208931 | MK208931 | – |
| 12 | *Microhyla karunaratnei* | DZ 1530 | Morningside, Sinharaja, Sri Lanka | – | MH807393 | – |
| 13 | *Microhyla kodial* | F1 | Mangaluru, Karnataka, India | – | MF919453 | – |
| 14 | *Microhyla laterite* | GL3303 | Manipal, Karnataka, India | KT600664 | KT600671 | – |
| 15 | *Microhyla malang* | MZB:Amp:16364 | Balikpapan, Kalimantan, Indonesia | AB634619 | AB634677 | – |
| 16 | *Microhyla marmorata* | KUHE:32455 | Xamneua, Houapan, Laos | AB634610 | AB634668 | – |
| 17 | *Microhyla mihintalei* | DZ1410 | Makandura, Sri Lanka | – | KU214857 | – |
| 18 | *Microhyla mukhlesuri* | ZMMU:NAP8311 | Myanmar | MK208934 | MK208934 | – |
| 19 | *Microhyla mymensinghensis* | MSP 04 | – | MK635478 | MK635493 | – |
| 20 | *Microhyla nilphamariensis* | MHLB00206 | Berakhuti, Barua, Nilphamari, Bangladesh | – | LC090057 | – |
| 21 | *Microhyla orientalis* | KUHE 55048 | Wongaya Gede, Bali, Indonesia | AB781454 | AB781465 | – |
| 22 | *Microhyla ornata* | ZSIK–A9119 | Dharwad, Karamana, India | AB201177 | AB201188 | – |
| 23 | *Microhyla palmipes* | MZB:Amp:16255 | Bedegul, Bali, Indonesia | AB634612 | AB634670 | – |
| 24 | *Microhyla petrigena* | KUHE:53743 | Bukit Kana, Sarawak, Malaysia | AB634617 | AB634675 | – |
| 25 | *Microhyla pulchra* |  | Dongguan, Guangdong, China | KF798195 | KF798195 | KF798195 |
| 26 | *Microhyla rubra* | ZMMU:A5006–19 | India | MK208936 | MK208936 | – |
| 27 | *Microhyla sholigari* | GL3360 | Biligirirangan Hills, Karnataka, India | KT600667 | KT600675 | – |
| 28 | *Microhyla taraiensis* | JRK201525 | Jamun Khadi, Jhapa,district, Nepal | MF496241 | MF496241 | MF496241 |
| 29 | *Microhyla zeylanica* | DZ 1419 | Horton plains, Sri Lanka | – | MH807428 | – |
| 30 | *Kaloula verrucosa* | – | Sichuan, China | MG962359 | MG962359 | MG962359 |
