# Supplementary figures and images for: Acoustic divergence in advertisement calls among three sympatric Microhyla species from East China
Source: PeerJ. 2020 Mar 11;8:e8708. doi: 10.7717/peerj.8708 (PMC7071819; doi:10.7717/peerj.8708)

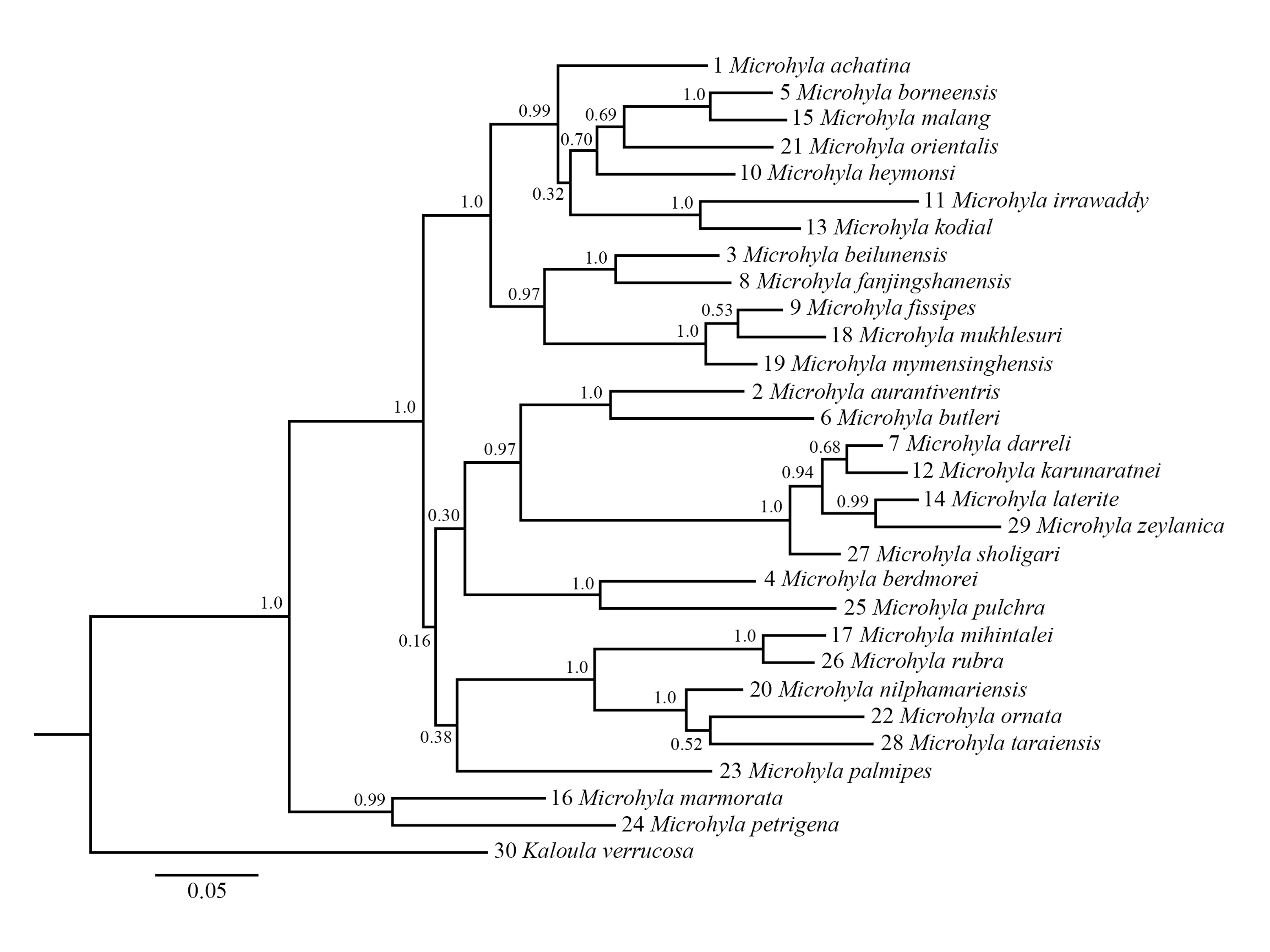

Supplement: Supplemental Information 3 — One Kaloula verrucosa sample is chosen as outgroup. Numbers on the nodes show posterior probabilities. The scaleplate represents substitutions/site. The specimen number and accession number are showed in appendix table 2. [file peerj-08-8708-s003.jpg]
